# Enhancing menaquinone-7 biosynthesis by adaptive evolution of *Bacillus natto* through chemical modulator

**DOI:** 10.1186/s40643-022-00609-0

**Published:** 2022-11-22

**Authors:** Bei Zhang, Cheng Peng, Jianyao Lu, Xuechao Hu, Lujing Ren

**Affiliations:** 1 College of Biotechnology and Pharmaceutical Engineering, Nanjing Tech University, No. 30 South Puzhu Road, Nanjing, 211816 People’s Republic of China; 2 Shanghai JanStar Technology Development Co., Ltd., No. 1288, Huateng Road, Shanghai, 201700 China

**Keywords:** Menaquinone-7, *Bacillus natto*, Adaptive evolution, Glyphosate, EPSE synthase

## Abstract

Menaquinone-7 (MK-7) is a kind of vitamin K2 playing an important role in the treatment and prevention of cardiovascular disease, osteoporosis and arterial calcification. The purpose of this study is to establish an adaptive evolution strategy based on a chemical modulator to improve MK-7 biosynthesis in *Bacillus natto*. The inhibitor of 5-enolpyruvylshikimate-3-phosphate synthase (EPSP synthase), glyphosate, was chosen as the chemical modulator to perform the experiments. The final strain ALE-25–40, which was obtained after 40 cycles in 25 mmol/L glyphosate, showed a maximal MK-7 titer of 62 mg/L and MK-7 productivity of 0.42 mg/(L h), representing 2.5 and 3 times the original strain, respectively. Moreover, ALE-25–40 generated fewer spores and showed a higher NADH and redox potential. Furthermore, the mechanism related to the improved performance of ALE-25–40 was investigated by comparative transcriptomics analysis. Genes related to the sporation formation were down-regulated. In addition, several genes related to NADH formation were also up-regulated. This strategy proposed here may provide a new and alternative directive for the industrial production of vitamin K2.

**Graphical Abstract:**

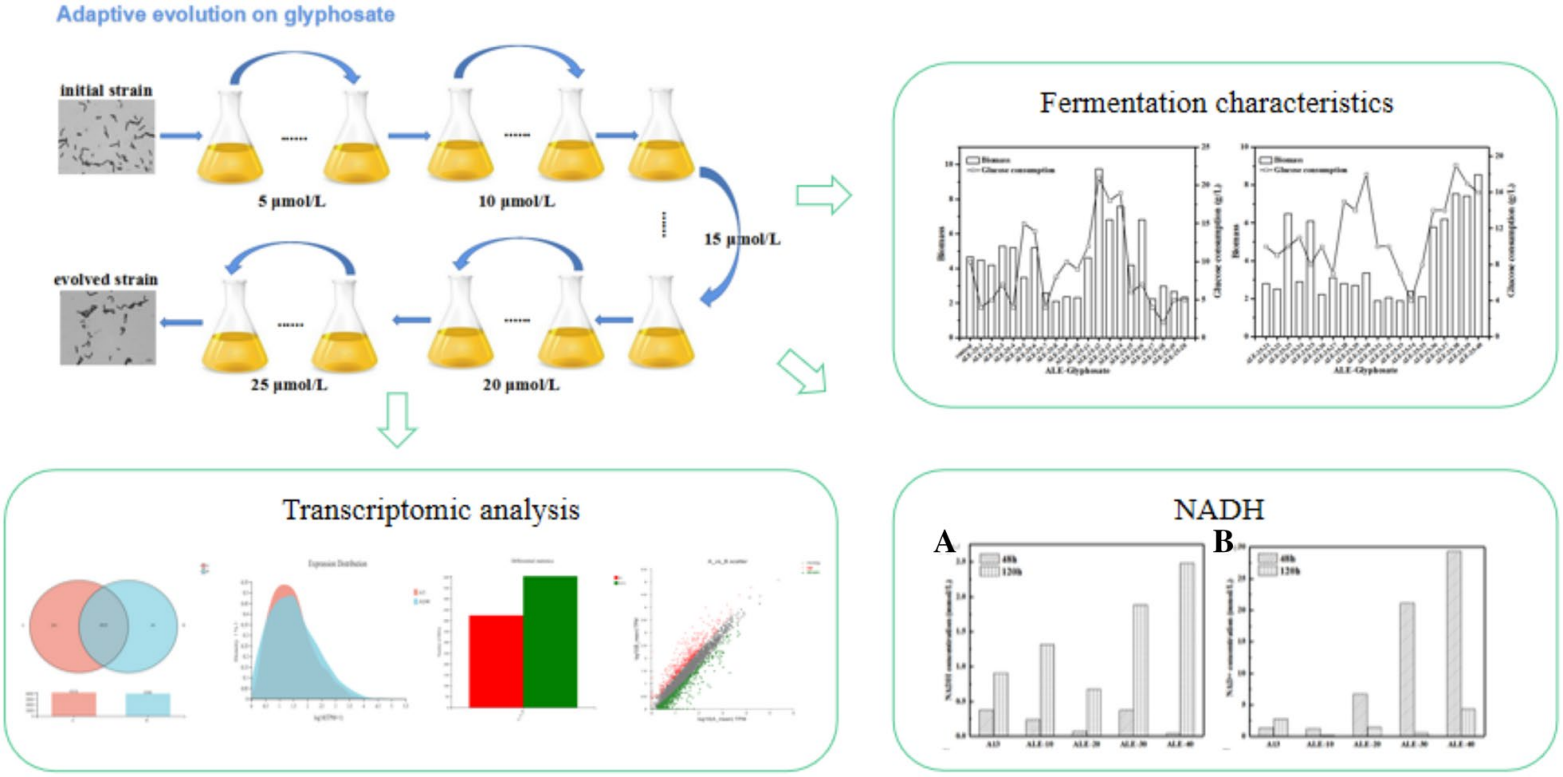

**Supplementary Information:**

The online version contains supplementary material available at 10.1186/s40643-022-00609-0.

## Introduction

Vitamin K2, also known as menaquinone (MK), is an essential fat-soluble vitamin, which plays an important role in the treatment and prevention of cardiovascular disease, osteoporosis and arterial calcification (Ren et al. 2020). It consists of 2-methyl-1,4-naphtoquinone nucleus and a side chain of isoprene units at the 3-position (Brudzynski and Flick 2019). Depending on the number of isoprene units, vitamin K2 is divided into 14 types, denoted by MK-*n* (*n* = 1,2…14) (Vermeer et al. 2017). *Bacillus subtilis natto*, a subspecies of *Bacillus subtilis*, is one of 40 kinds of edible probiotics recognized by Food and Drug Administration (FDA) in the USA (Sella et al. 2021). Because of its fast growth, high content of MK-7 and safety certification, it is considered to be the most potential strain for industrial production of MK-7.

Until now, many efforts have been devoted to improve the MK-7 yield. For example, Liu et al. proposed a high-throughput screening strategy based on the fluorescence-activated cell sorting, which could convert the MK content to a fluorescence signal and greatly improved the screening efficiency (Liu et al. 2016). Furthermore, using the precursor of menaquinone-7,1,4-dihydroxy-2-naphthalate (DHNA), as the selector to screen high MK producing strain, Wu et al. established an improved gradient radiation breeding strategy based on low energy N^+^ ion implantation(Wu et al. 2021). The fermentation processes were also optimized including changing the cultivation method (Hu et al. 2017), increasing the redox potential (Wang et al. 2019) or enhancing the MK-7 secretion (Zhao et al. 2021). Meanwhile, the menaquinone biosynthesis pathways were also transferred to different model microorganisms, such as *E. coli* (Gao et al. 2020), yeast (Jun et al. 2020), and *Bacillus subtilis* (Yang et al. 2020), which greatly improved the MK-7 yield. However, the MK-7 industrial production is still restricted by low productivity. Therefore, it is still important and necessary to develop more effective and safe technologies to improve the strain performance and thus increase MK-7 point production.

Adaptive evolution is an effective way to improve the performance of strains (Wu et al. 2022). Under certain stress conditions, microorganisms could enrich the favorable genetic changes brought by the specific pressure during the successive cultivation (Espinosa et al. 2020). Liu et al. obtained a high-temperature resistant *Bacillus amyloliquefaciens* through long-term high-temperature cultivation and the MK-7 yield of the strain was improved by 2 times at 50 ^°^C using starch as the sole carbon source (Liu et al. 2021). Besides, a sequential cultivation of the C5-utilizing *Bacillus coagulans* with ethanol resulted in 2.6-fold increase in lactic acid yield (Cubas-Cano et al. 2020). Yuan et al. found lipase production was coupled to the tributyrin uptake rate, then they cultivated *B. subtilis* SPZ1 using tributyrin as the sole carbon source for over 1000 generations, finally the lipase activity was improved by 1.9-fold (Yuan et al. 2019). In addition to using the nutritional and environmental stress as the pressures, chemical regulators are also good pressures (Im et al. 2013). Chemical regulators are chemical substances that can interfere with intracellular metabolism by specifically targeting enzymes or acting as signal molecules (Maeda et al. 2019). In the example of lipid production, Diao et al. acclimated marine microalgae for more than 800 days with sethoxydim and sesamol, the inhibitors of acetyl-CoA carboxylase and malic enzyme. Finally, the total lipid content increased by 100% (Diao et al. 2019). Therefore, adaptive evolution can be used as a powerful tool to improve the strain performance and increase the final product yield.

In this study, the chemical regulator glyphosate, an inhibitor of 5-enolpyruvylshikimate-3-phosphate synthase (EPSP synthase) (Hertel et al. 2021), was selected as selective pressure to perform the adaptive evolution on *Bacillus natto*. EPSP synthase is the key enzyme of shikimate pathway in MK-7 biosynthesis (Johnston and Bulloch 2020). First, the effect of different glyphosate concentrations on cell growth and MK-7 biosynthesis was studied. Then, the initial strain was continuously cultivated in the gradient concentration of glyphosate until getting the evolved strain of ALE-25–40. Next, the fermentation performance of different evolved strains was compared including biomass, glycerol consumption, MK-7 yield, and cell morphology. Furthermore, the comparative transcriptomics coupling with the redox potential and the NADH/NAD^+^ ratio was analyzed between the evolved and wild strains to decipher the underlying metabolic mechanism.

## Materials and methods

### Microorganism, and culture conditions

*Bacillus natto* A13 (CCTCC M 2,014,405) was used as the starting strain in this study. The evolved strain *Bacillus natto* ALE-25–40 (GDMCC NO. 61234) was stored in the Guangdong microbial culture collection center. The seed medium and culture conditions was same as our previous study (Luo et al. 2016). The seed was stored in 20% glycerol at − 80 ^°^C. After three generations in the seed medium for 12 h at 37 ^°^C, 200 rpm, the seed culture was transferred to 500 mL shake flask containing 100 mL fermentation medium with glycerol 60 g/L, soybean peptone 100 g/L, yeast powder 0.6 g/L, K_2_HPO_4_·3H_2_O 0.3 g/L, CaCl_2_ 0.1 g/L, MgSO_4_·7H_2_O 0.3 g/L. Cells were cultivated for 6 days at 37 ^°^C, 200 rpm.

### Adaptive laboratory evolution experiments

Before performing the adaptive evolution experiments, different concentrations (5, 10, 15, 20, 25 μmol/L) of glyphosate were added to the seed medium to study the inhibition of cell growth and MK-7 biosynthesis. The inhibition ratio was calculated by the difference in biomass of the control and the experimental group divided by the OD of the control group:

$$\mathrm{Inhibition \,rate}=\frac{{\mathrm{OD}600}_{\mathrm{control}}-{\mathrm{OD}600}_{\mathrm{experimental}}}{{\mathrm{OD}600}_{\mathrm{control}}}\times 100\%$$

Then, ALE was performed using a long-term serial transfer procedure. The glyphosate concentration increased by 5 μmol/L every 5–10 generations from 5 μmol/L and then kept constant until 25 μmol/L. Forty generations were cultivated at the medium with 25 μmol/L glyphosates. During the cultivation, cells will transfer to the solid medium with glyphosate to purify the strains every 10 generations. Four evolved strains of ALE-25–10, ALE-25–20, ALE-25–30, and ALE-25–40 were obtained.

### Analysis of glucose, glycerol, biomass, and cell morphology

Glucose concentration was detected by the SBA-40C glucose biosensor. The residual glycerol was detected by a free glycerol determination kit (Sigma–Aldrich Co. USA). The fermentation broth was diluted to a suitable concentration, and the OD600 value was determined by UV spectrophotometer (UV-1200, Shanghai, China). Cells were stained with crystal violet and observed under a light microscope (Leica DM1000, Leica Microsystems, Morrisville, NC, USA).

### Extraction and analysis of MK-7

The MK-7 yield was measured through the method used in our previous study (Ma et al. 2019). 1 mL broth was mixed with 2 mL extraction agent (*N*-hexane:isopropanol = 2:1) and high-speed blended for 30 min. Then, the mixture was centrifuged at 10,000 rpm for 5 min. The supernatant was evaporated to get the yellow oily substrates and redissolved in the methanol for the further HPLC analysis.

### Determination of redox potential, NADH and NAD^+^ concentration

The intracellular NADH and NAD^+^ content was determined by the spectrophotometric method using the NAD(H) detection kit (Suzhou Comin Biotechnology, China). The redox potential of the broth was detected every 24 h by the REDOX potentiometer (Beijing Shunkeda Technology Co., LTD, China).

### Transcriptomics analysis

After 48 h of cultivation, cells of the starting strain and the evolved strain ALE-25–40 were collected. RNA extraction, RNA sequencing, gene function annotation and differential expression analysis were performed in the same way as in our previous study (Peng et al. 2020).

## Results and discussion

### Growth properties variation of *Bacillus natto* during adaptation

**Fig. 1:**
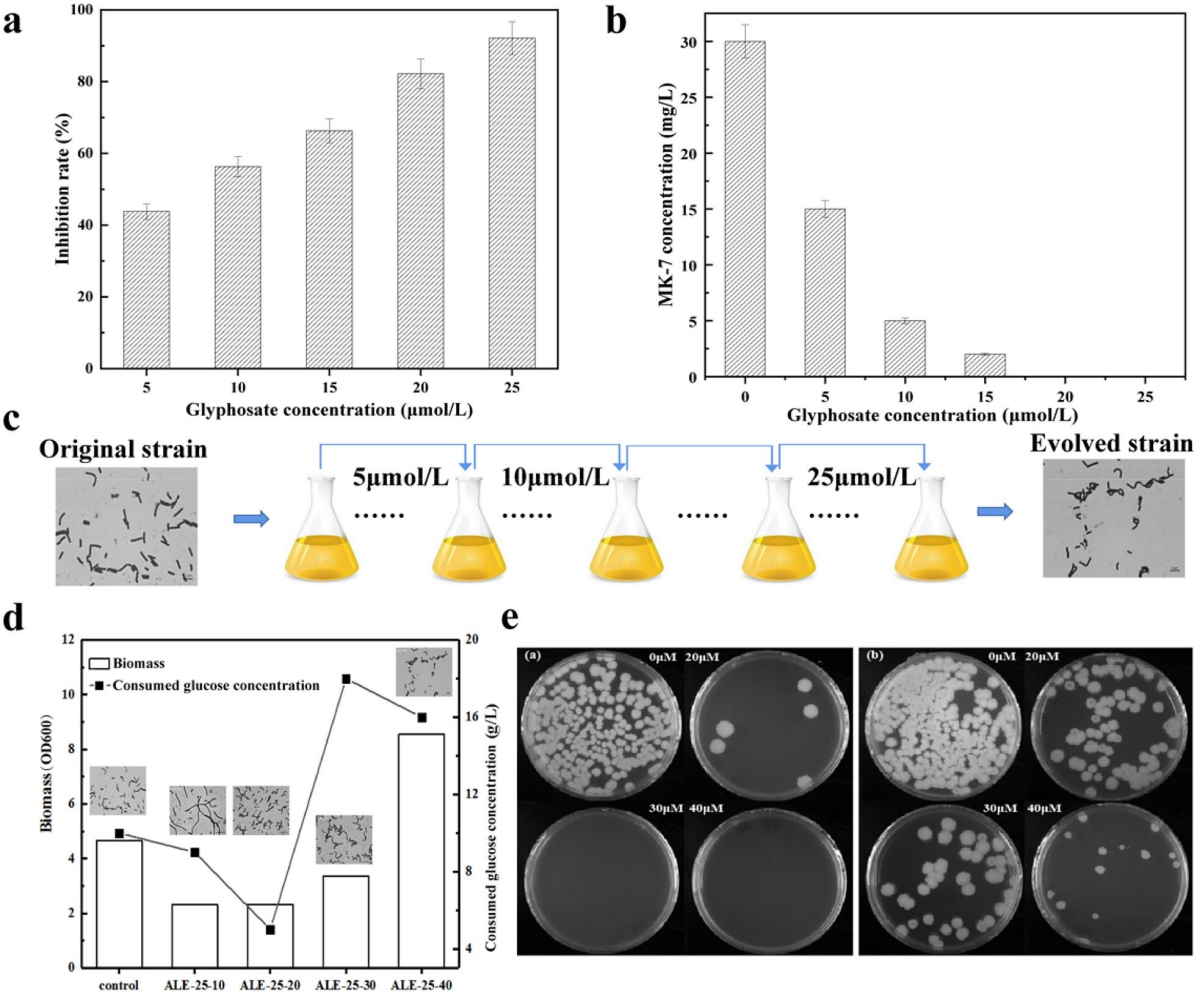
Changes of cell growth properties during adaptation. Cell inhibition rates (**a**) and MK-7 concentration (**b**) at different glyphosate concentrations; (**c**) adaption experiment flow; (**d**) changes of biomass and consumed glucose concentration during adaption; (**e**) glyphosate tolerance of original strain (**e**-**a**) and evolved strain (**e**-**b**)

As the inhibitor of EPSP synthase, glyphosate would affect cell growth obviously. The cell growth inhibition rate was 43.8% when glyphosate was added at 5 μmol/L, and the concentration of MK-7 decreased from 30 mg/L when glyphosate was not added to 15 mg/L (Fig. 1a, b). The inhibitory effect was more obvious as the increase of glyphosate concentration. The inhibition rate reached more than 90% with 25 μmol/L glyphosates and there was no MK-7 accumulation at this condition. Next, we started the adaptive evolution experiment from 5 μmol/L glyphosates and increased the concentration when cells adapted the new environment until 25 μmol/L, then kept at this concentration for 40 generations (Fig. 1c), generating four evolved strains of ALE-25–10, ALE-25–20, ALE-25–30, and ALE-25–40.

As shown in Fig. 1d, the value of OD_600_ for the ALE-25–10 and ALE-25–20 strains decreased by 50.5–50.0%, when compared with that of the control, but the biomass of ALE-25–40 not only reached the control level, but also increased, demonstrating that the strain gradually adapted to glyphosate environment. Meanwhile, the consumed glucose showed similar trends. When comparing the cell morphology of the four strains during the adaption, we found only ALE-25–10 displayed a different state with a long rod shape, implying the cell grew slowly at this condition and was still in the early stage of cell growth. Further detecting the glyphosate tolerance of ALE-25–40 at the solid medium with 20, 30 and 40 μmol/L glyphosates, we found the starting strain cannot grow at 30 μmol/L but the ALE-25–40 could still grow well at 40 μmol/L, indicating the glyphosate tolerance of the evolved strain had indeed improved.

### Fermentation characteristics changes of ALE strains

**Fig. 2:**
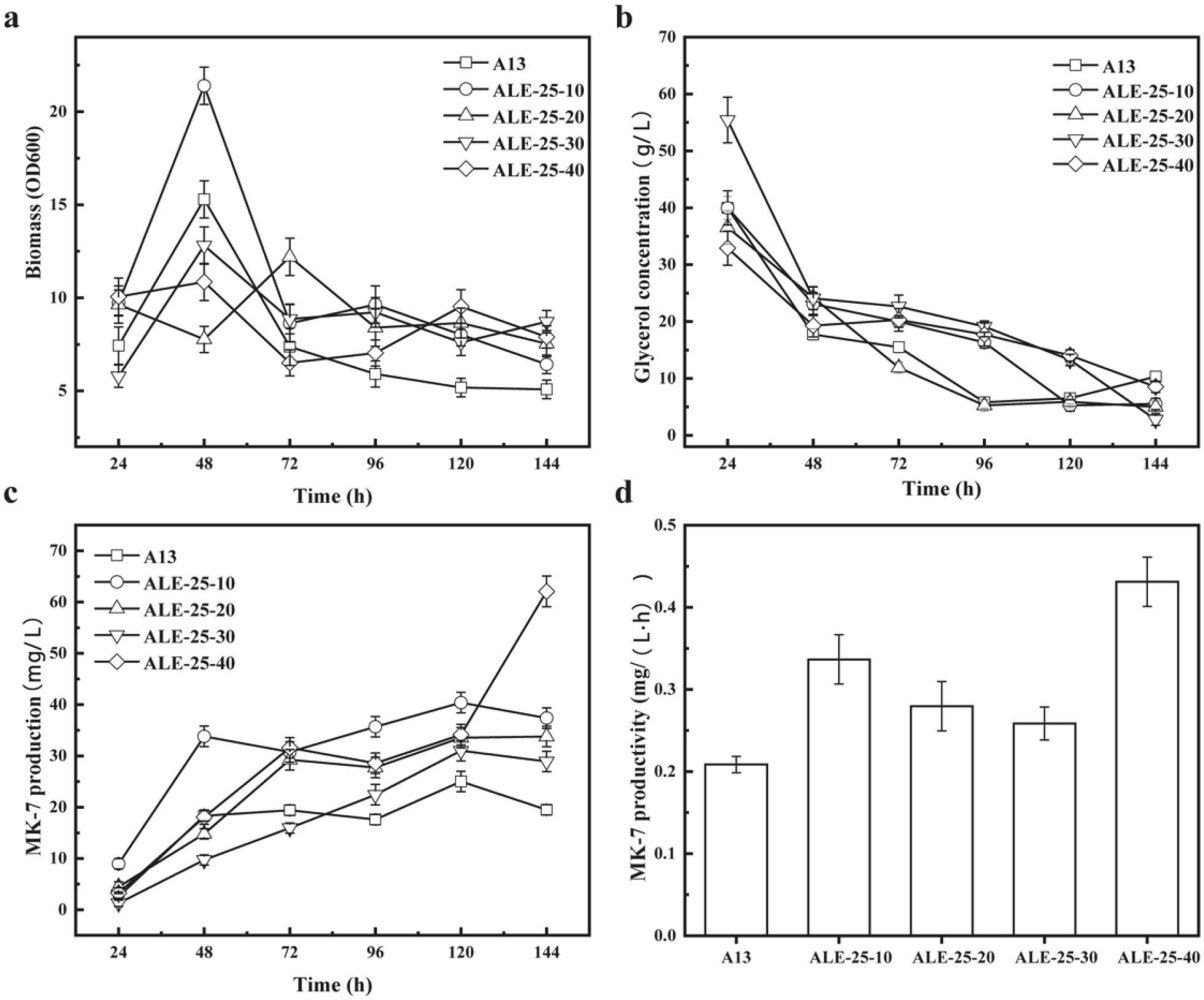
Comparison of cell growth (**a**), glycerol concentration (**b**), MK-7 production (**c**), and MK-7 productivity (**d**) of different adaptive strains and original strain

In an attempt to evaluate the productivity of different evolved strains, we investigated the changes in the fermentation characteristics between the starting strain and the evolved strains ALE-25–10, ALE-25–20, ALE-25–30, and ALE-25–40, cultivated under the same conditions at 37 ^°^C in the normal fermentation medium. As shown in Fig. 2a, the evolved strains did not show a significant growth advantage at the early stage but the OD_600_ values of all the evolved strains after 96 h were all higher than that of the starting strain. Further comparing the morphology of these strains, we found ALE-25–40 generated fewer spores at the late stage of the fermentation (Additional file 1: Fig. S1), and most of the strains still kept rod-shaped, implying that the evolved strains might possess less ability to form spores.

However, the glycerol consumption rate of the control was the highest. As shown in Fig. 2b, the glycerol concentration of the starting strain was exhausted at 96 h, while the timepoint of glycerol depletion of the evolved strain was extended to 144 h, demonstrating that the glycerol consumption rate of the evolved strains slowed down obviously. Further comparing the MK-7 yield of these strains, the MK-7 yield of all the evolved strains was higher than that of the control. Although the evolved strain ALE-25–40 had no obvious advantage at the beginning of fermentation, MK-7 yield still kept increasing and improved greatly after 120 h, and the final MK-7 yield of ALE-25–40 reached 62 mg/L, which was 2.5 times the value of control (Fig. 2c). From Fig. 1d, it can be seen that fewer spores were generated in ALE-25–40 at the late stage of fermentation and this good status of the cell might be one of the reasons for the higher yield of ALE-25–40. Besides, the MK-7 productivity of ALE-25–40 reached 0.42 mg/(L·h), which was 3 times he value of the control (Fig. 2d).

The above results demonstrated that the long-time adaptation in the glyphosate environment could indeed improve the MK-7 yield and productivity without affecting cell growth rate obviously but decreased the sporulation process. It is reported that spore formation would slow down or stop the MK-7 biosynthesis (Berenjian et al. 2014), this might explain the phenomenon that the MK-7 yield of evolved strain could keep increasing in the later stage, while the yield of the control strain remained stable after 72 h. MK-7 could be regarded as the secondary metabolite, so the cell growth and product accumulation were not positively correlated. Some people also call them the tradeoff phenomenon (Dragosits and Mattanovich 2013). For example, Sun et al. cultivated *Schizochytrium sp*. continuously in a high-oxygen environment, and the cell dry weight was increased by 32.4%, but the final lipid content was decreased (Sun et al. 2016).

### Changes of NADH/NAD^+^ and redox potential of the evolved strain

**Fig. 3:**
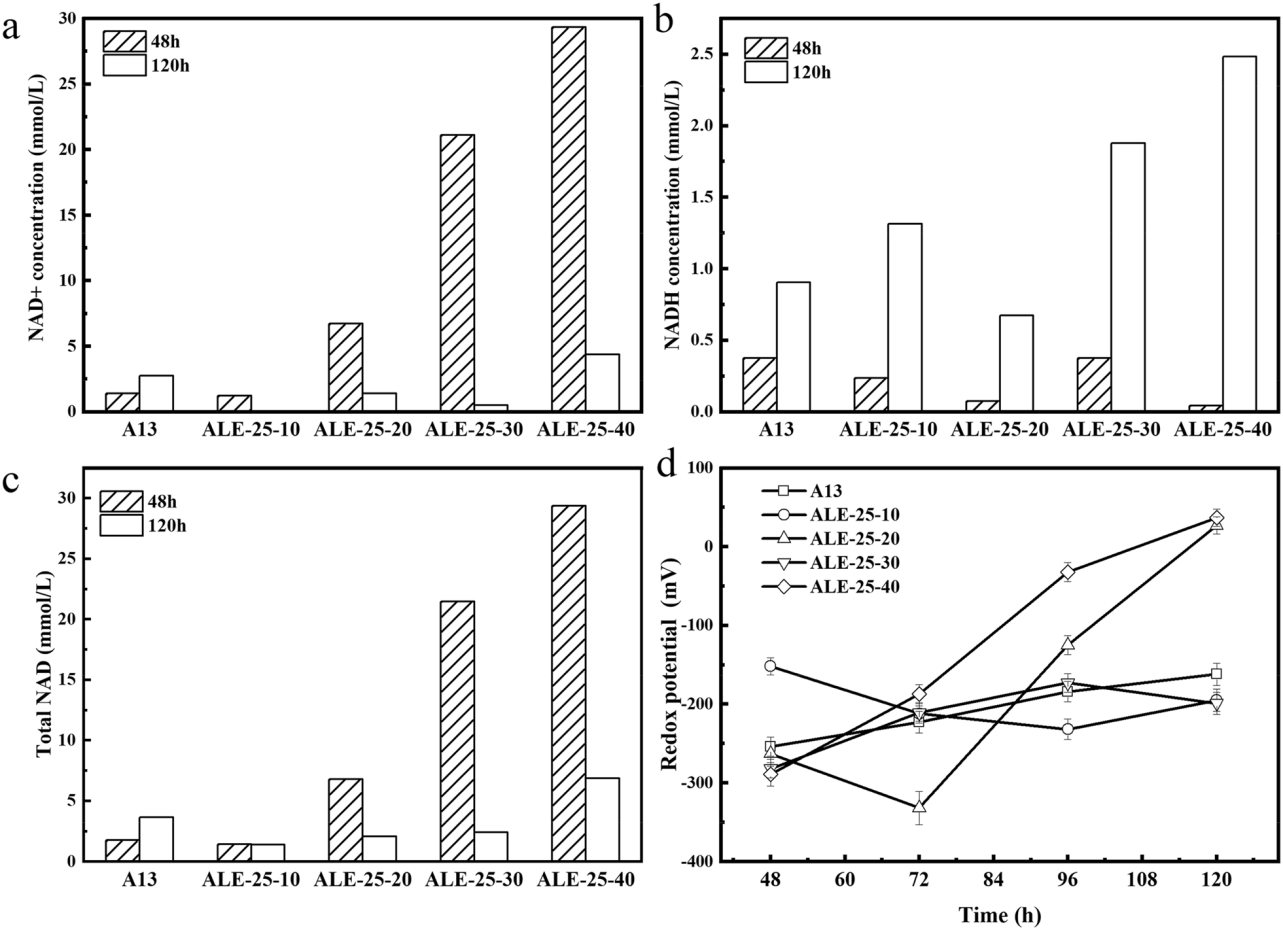
Comparison of NADH (**a**), NAD^+^ (**b**), total NAD (**c**) concentrations and redox potential (**d**) of different adaptive strains and original strain

To further analyze the underlying mechanism of the titer improvement of adapted strains, the REDOX potential and the ratio of NADH/NAD^+^ were compared between wild type and adapted strains. NADH and NAD^+^ are important coenzymes in microorganisms which could store energy and will be converted to ATP when needed (Zhao and Yang 2015), so the intracellular NADH and NAD^+^ contents were also detected to reflect the changes in the intracellular energy metabolism. As shown in Fig. 3a, there was no big difference in NADH concentration at 48 h, but the value at 120 h were all higher than that of the A13 strain except ALE-25–20. In addition, the changes in NAD^+^ and the total NAD concentration showed the opposite trend (Fig. 3b, c). The NADH concentration of ALE-25–10, ALE-25–30 and ALE-25–40 at 120 h were 1.5, 2.1 and 2.7 times that of A13, respectively. The NAD^+^ and total NAD concentration of ALE-25–40 at 48 h were 21.12 and 16.66 times that of the original strain, respectively. This might be due to the fact that long time cultivation in the medium with glyphosate might not only promote the shikimic acid biosynthesis, but also enhanced the overall metabolic activity.

In addition, NADH is the electron donor of the electron transport chain (Godde and Trebst 1980). Menaquinone-7 acts as the electron transfer carrier. The faster the electron transfer, the more MK-7 is needed, thus the synthesis of MK-7 is significantly enhanced. Cui et al. increased MK-7 titer in a 15L bioreactor by 55% to 310 mg/L by co-expressing the cell membrane components signals transduction protein tatAD-CD and the cytochrome c reductase qcrA-C (Cui et al. 2020). The higher NADH concentrations of the adaptive strains at 120 h implied that more NADH would enter the electron transport chain and activate the electron transport and need more menaquinone. This might be one of the reasons for the increased MK-7 production of the adaptive strains. Similar to MK-7, coenzyme Q10 is also an electron transporter in the respiratory chain. Zhu et al. increased CoQ10 production by 58% through overexpression of glyceraldehyde-3-phosphate dehydrogenase (Zhu et al. 2017). In addition, Xu et al. increased squalene titers by 59% by expressing pyridine nucleotide transhydrogenase (udhA) to improve the cellular NADPH/NADP^+^ ratio (Xu et al. 2019). These examples demonstrated a positive relationship between the cellular NADH or NADPH concentration and different biochemical production, which could also explain the enhancement of MK-7 in this study.

As the two dominant redox pairs in the cytoplasm, the ratios of NADH/NAD^+^ determine the intracellular oxidation–reduction potential (ORP) (Braissant et al. 2020). The ORP level could reflect the macroscopic REDOX properties of the fermentation broth. The higher the REDOX potential is, the stronger the oxidability is, and the lower the REDOX potential is, the stronger the reducibility is. As shown in Fig. 3d, the ORP level at 48 h all kept between − 290 mV and − 250 mV except ALE-25-10 which reached − 160 mV, indicating that all strains grew in a relative reductive environment at the early stage of fermentation. The change in ORP level during fermentation varied among these five strains. The REDOX potentials of ALE-25-20 and ALE-25–40 increased significantly, reaching 27 mV and 37 mV, respectively, at 120 h, while the changes of other strains were not obvious, remaining from − 200 mV to − 160 mV. It is worth noting that the REDOX of ALE-25–40 kept increasing during the fermentation which might be related to the higher MK-7 concentration, implying that the MK-7 yield might be positively correlated with REDOX potential and the reductive environment might be more conducive for MK-7 accumulation.

In addition, many studies have also explored the relationship between product accumulation and REDOX potential. The optimal REDOX potential varies for different microorganisms (Ren et al. 2006). It is reported that the cultivation environment with strong reducibility was more conducive to the conversion of glycerol to ethanol, propanediol and other reducing products. In addition, Riondet et al. changed the ratio of formate by regulating the REDOX potential of *E. coli* and they found the changes of extracellular REDOX potential could regulate the activity of some specific enzymes, thus changing metabolic flux, ATP production and product synthesis (Riondet et al. 2000). For MK-7 production, the greater the changes of REDOX potential during fermentation, the higher the yield of MK-7.

### Comparative transcriptomic analysis of the evolved strain

In addition, we analyzed transcriptomic data to elucidate the regulatory mechanisms responsible for changes in cell growth and MK-7 production in ALE-25–40. To explore the metabolic pathway changes in ALE-25–40, functional annotation of differentially expressed coding genes was performed.

#### Sporulation

**Fig. 4:**
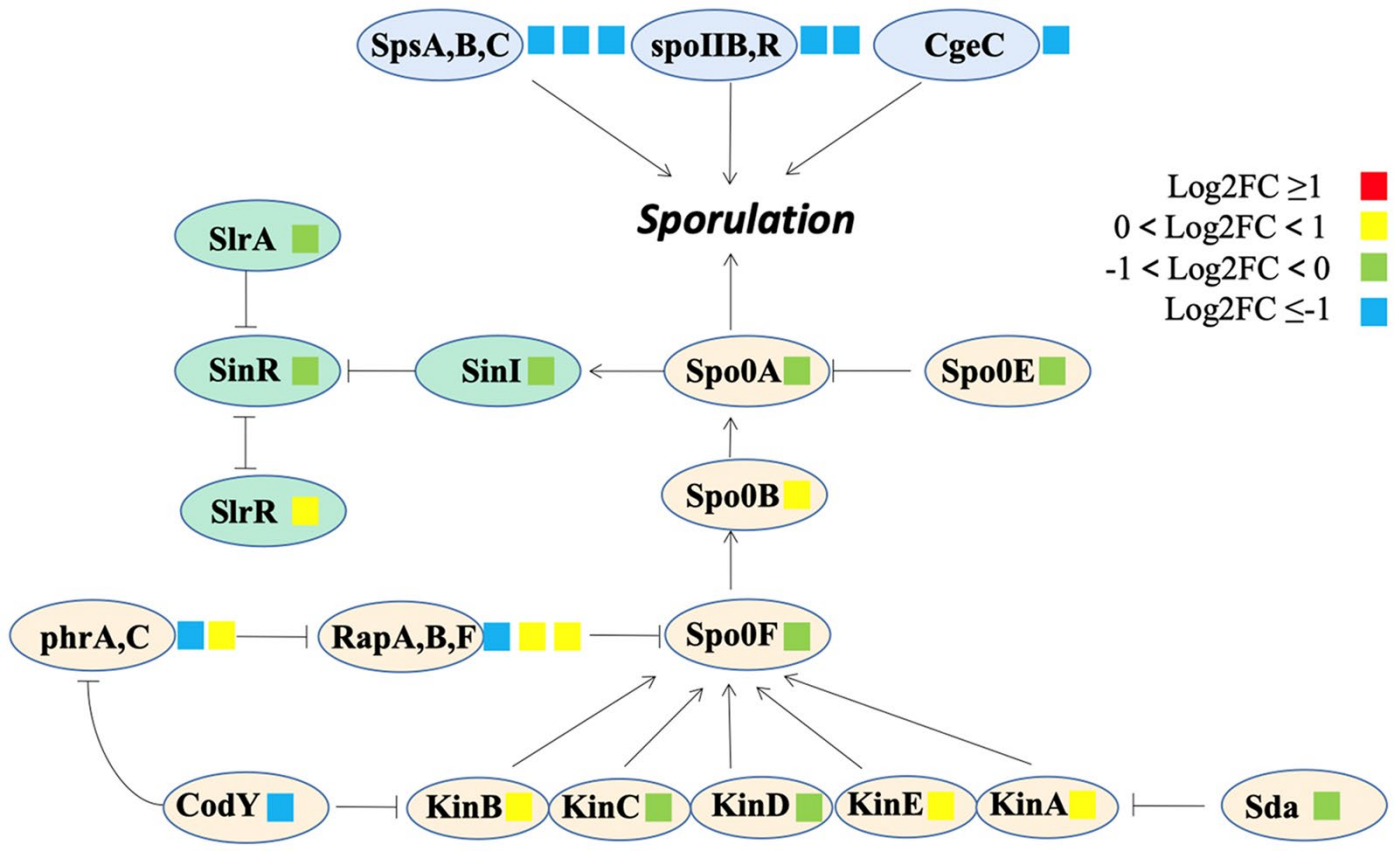
Changes in transcript abundance of genes involved in sporulation in ALE-25–40

Spore formation is the characteristic of *Bacillus subtilis* and will slow down or stop the MK-7 biosynthesis (Berenjian et al. 2014). Most of the genes related to the sporulation were down-regulated in ALE-25–40 (Fig. 4). spo0A, the main regulator of sporulation and could promote spore formation by phosphorylation (Molle et al. 2003), was down-regulated by 0.21-fold. Besides, several regulation genes related to spo0A in the sporulation phosphorelay system were also down-regulated. The phosphatase rapA and its active secretion inhibitor phrA and transcriptional regulator codY were down-regulated by 0.72-, 0.75-, and 0.81-fold, respectively. In addition, the protoplasm of the spore is surrounded by three membranes including a thick cortex, spore coat, and spore envelope (Hashimoto and Naylor 1958). We also found that several spore coat polysaccharide biosynthesis proteins including spsA, spsB, spsC, etc. were also down-regulated. The second stage spore formation proteins of spoIIB and spoIIR and the spore maturation protein of cgeC were also found down-regulated by 0.58-, 0.87-, and 0.61-fold, respectively. The thick cortex is composed of peptidoglycan and the spores coat is part of the thick cortex (Imamura et al. 2011), which is associated with the synthesis of the spores coat. The spores coat consists of proteins containing large amounts of cysteine in a laminar structure. cgeC is the protein involved in the maturation of the outermost layer of the spore, that is, the outermost material outside the spores coat (Imamura 2012). All these genes are involved in regulating the process of spore formation itself, and their down-regulation indicates that the spores are not mature or that there are particularly few mature spores. This may be because the long-term domestication of glyphosate might make the strain stronger. Adaptive strains generated fewer spores than the original strains in the same harsh environment, and it was consistent with the phenotype of fewer spores in the fermentation process. Cui et al. improved MK-7 biosynthesis through inhibiting spore formation by knocking out spoIIA and spoIIE (Cui et al. 2019). Our previous study also proved that most of the genes related to sporulation were down-regulated at the condition with high MK-7 yield (Peng et al. 2020). These studies proposed the similar findings and could provide theoretical support for this study.

#### Central carbon metabolism

**Fig. 5:**
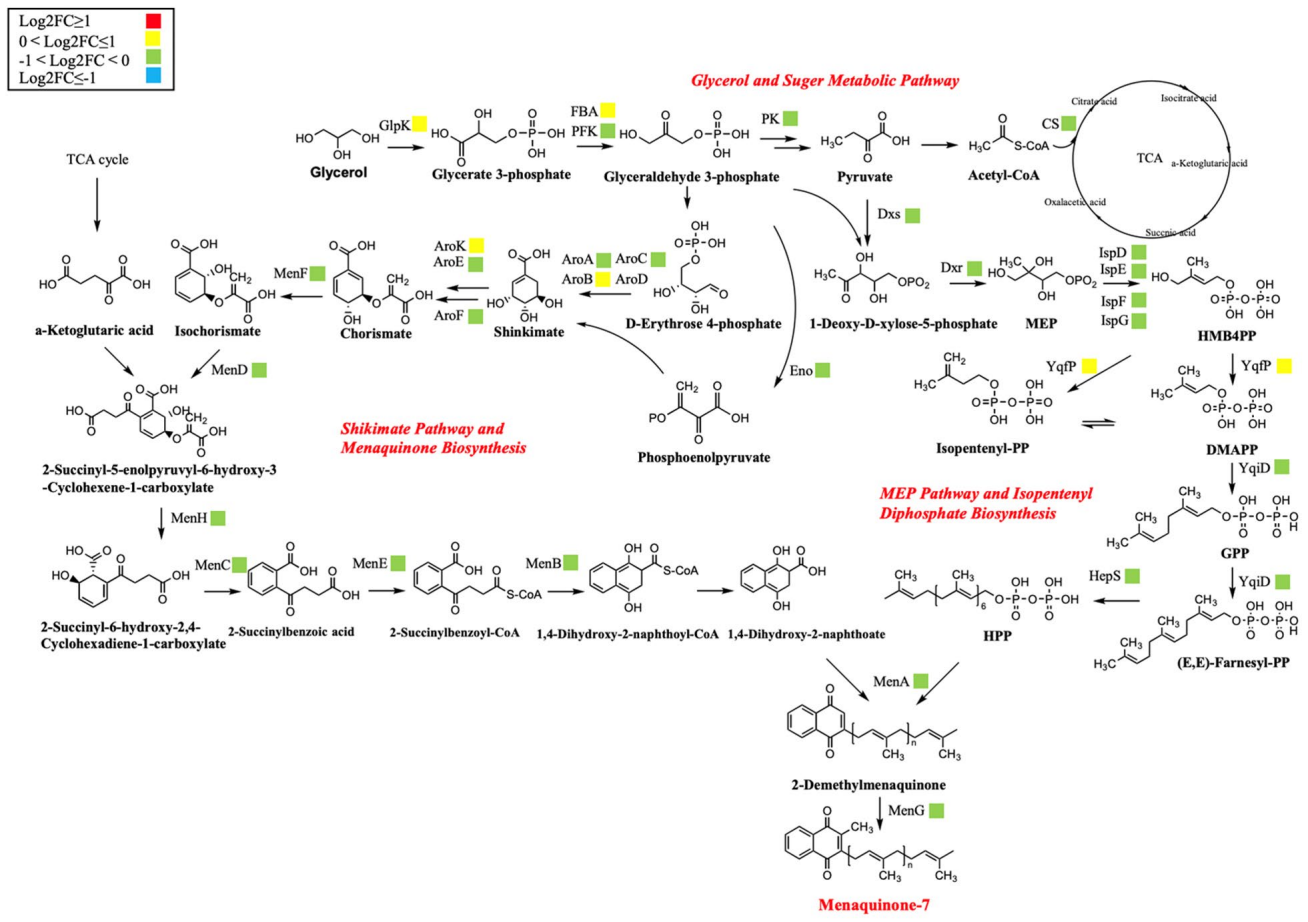
Changes in transcript abundance of genes involved in menaquinone-7 metabolic pathway in ALE-25–40

The biosynthetic pathway of MK-7 within *Bacillus subtilis* consists of the glycolytic pathway (EMP), the pentose phosphate pathway (HMP), the 2-methyl-d-erythritol-4-phosphate pathway (MEP) and the menaquinone synthesis pathway (MK) (Yang et al. 2019). The transcriptome analysis (Fig. 5) showed that domestication did not have a significant effect on the biosynthetic pathway of the strain itself, with only five genes, glpK, FBA, aroB, aroK and yqfP, being more significantly up-regulated. glpK acts as an enzyme for the conversion of glycerol to 3-phosphoglycerol, which is then converted to 3-phosphoglycerol. AroB and aroK are two enzymes for the chorismate biosynthesis. Yafp is the key enzyme during the formation of isoprene side chain. The upregulation of these genes would be beneficial for MK-7 biosynthesis.

#### Antioxidant defense system

**Fig. 6:**
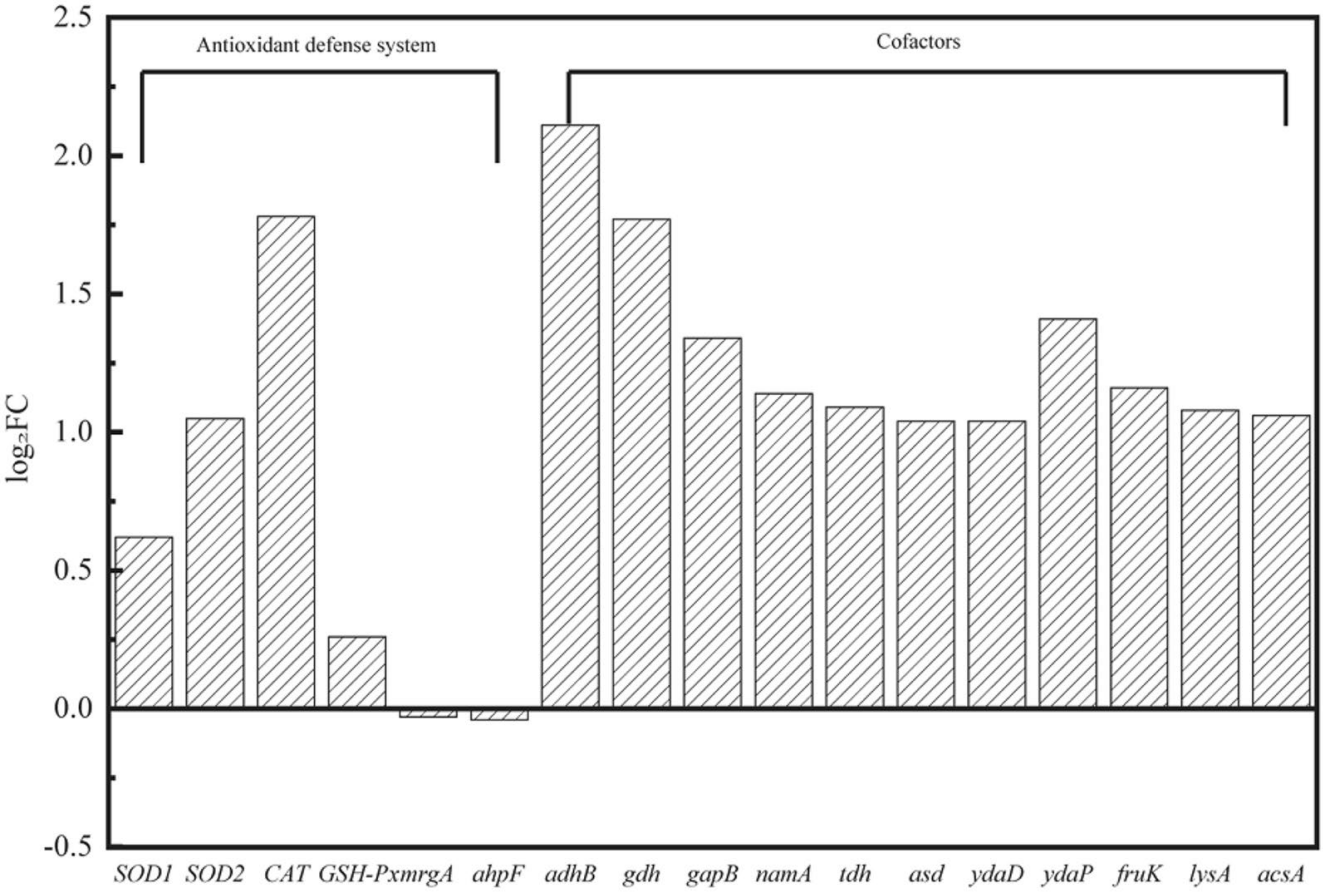
Changes in transcript abundance of genes involved in antioxidant defense system and NADH generating enzymes in ALE-25–40.* SOD1/SOD2* superoxide dismutase, *CAT* vegetative catalase 1, *GSH-Px* glutathione peroxidase, *mrgA* metalloregulation DNA-binding stress protein, *ahpF* alkyl hydroperoxide reductase large subunit and NADH dehydrogenase, *adhB* alcohol dehydrogenase, *gdh* glucose-1-dehydrogenase, *gapB* glyceraldehyde-3-phosphate dehydrogenase, *namA* NADPH dehydrogenase namA, *tdh* l-threonine 3-dehydrogenase, *asd* aspartate–semialdehyde dehydrogenase, *ydaD* short-chain dehydrogenase, *ydaP* pyruvate oxidase, *fruK* fructose-1-phosphate kinase, *lysA* diaminopimelate decarboxylase, *acsA* acetyl-CoA synthetase

Reactive oxygen species (ROS) are often generated in cells under stress conditions, thus leading to cell damage (Jurkovic et al. 2008). Long time adaptation in glycerate conditions might improve the antioxidant defense system. Five genes related to antioxidant defense system including superoxide dismutase (SOD), catalase (katA), glutathione peroxidase (GSH-px), alkyl hydroperoxide reductase (ahpF) and DNA binding protein (mrgA), were compared to explore the effect of adaption on cell defense and MK-7 production (Zhu et al. 2020). As shown in Fig. 6, SOD1, SOD2, CAT, and GSH-Px were all up-regulated, in which SOD2 and CAT were most significantly up-regulated by 0.97- and 2.28-fold, respectively. Superoxide dismutase 2 (SOD2) is essential in radical scavenging and could balance the intracellular level of reactive oxygen species (ROS) (Wang et al. 2017). SOD2 is considered to be the first line of defense against ROS (Ruenwai et al. 2011) and can catalyze the disproportionation of superoxide anion radicals to generate O_2_ and H_2_O_2_. The apparent up-regulation of SOD2 demonstrates that a large amount of H_2_O_2_ might be produced during domestication. However, CAT plays an important role in the elimination of ROS (Dowds 1994), breaking down the cell-damaging H_2_O_2_ into O_2_ and H_2_O. This also demonstrated that domestication might indirectly increase the oxygen level during cell growth, which was beneficial for MK-7 biosynthesis as our previous study proved (Ma et al. 2019).

#### Cofactors formation

Considering the significant differences in NADH and NAD^+^ concentration of ALE-25–40 and A13, we further screened key genes associated with coenzyme formation. Most of them were significantly up-regulated. As shown in Fig. 6, alcohol dehydrogenase (adhB) and glucose-1-dehydrogenase (gdh) were up-regulated by 3.12- and 2.24-fold, respectively. In addition, glyceraldehyde-3-phosphate dehydrogenase (gapB), fructose-1-phosphate kinase (fruK) and NADPH dehydrogenase (namA) were up-regulated by 1.41-, 1.12- and 1.09-fold, respectively. The reactions involved in alcohol dehydrogenase and glucose-1-dehydrogenase all catalyze the formation of NADH by binding H^+^ of the compound to NAD^+^. NADPH dehydrogenase is an important oxidoreductase that is involved in a variety of physiological processes and biochemical metabolism. NADPH is the product of electron acceptance by the final electron acceptor NADP^+^. NADPH is usually used as a reducing agent for biosynthesis and does not enter the respiratory chain directly to be oxidized. It is only under the action of NADPH dehydrogenase that H^+^ on NADPH is transferred to NAD^+^ and then enters the respiratory chain as NADH (Kaur et al. 2018). l-Threonine-3-dehydrogenase (Zhang et al. 2019), aspartate semialdehyde dehydrogenase (Dahal and Viola 2015) and short-chain dehydrogenase (Alenazi et al. 2022) could regulate cellular oxidation and have the same function as the dehydrogenases mentioned above, increasing the intracellular NADH levels together. These could also explain the higher NADH level of the adaptive strains in 3.3 (Fig. 4b).

## Conclusions

In this study, the adaptive strain *Bacillus natto* ALE-25–40 was obtained through 40 cycles of adaptive evolution in the medium with 25 mmol/L glyphosates. It was demonstrated that this strategy based on a chemical modulator could be used to construct an adaptive strain with fewer spores and high menaquinone-7 productivity. Further comparative transcriptomics analysis showed that the enhanced performance of ALE-25–40 was mostly related to less spore formation and higher NADH generation. Moreover, in the evolved strain, genes of the antioxidant capacity were also upregulated.

### Supplementary Information

**Additional file 1:** **Figure S1.** Spore diagram of original strain (left) and ALE-25–40 (right) at the late stage of the fermentation. **Figure S2.** Venn diagram of the number of genes expressed in the original and domesticated strains. **Figure S3.** Number of up- and down-regulated differentially expressed genes in domesticated strains compared to the original strain. **Figure S4.** Volcano plot of expression difference between original and domesticated strains. **Table S1.** Differentially expressed genes associated with MK-7 biosynthesis. **Table S2.** Differential expression of key genes associated with spore formation. **Table S3.** Differential expression of key genes related to antioxidant defense system.

## Data Availability

All data generated or analyzed during this study are included in this published article.
