# Supplementary material for: Enhancing menaquinone-7 biosynthesis by adaptive evolution of Bacillus natto through chemical modulator
Source: Bioresour Bioprocess. 2022 Nov 22;9(1):120. doi: 10.1186/s40643-022-00609-0 (PMC10992315; doi:10.1186/s40643-022-00609-0)
Supplement: Supplementary file 1 — Additional file 1: Figure S1. Spore diagram of original strain (left) and ALE-25–40 (right) at the late stage of the fermentation. Figure S2. Venn diagram of the number of genes expressed in the original and domesticated strains. Figure S3. Number of up- and down-regulated differentially expressed genes in domesticated strains compared to the original strain. Figure S4. Volcano plot of expression difference between original and domesticated strains. Table S1. Differentially expressed genes associated with MK-7 biosynthesis. Table S2. Differential expression of key genes associated with spore formation. Table S3. Differential expression of key genes related to antioxidant defense system. [file 40643_2022_609_MOESM1_ESM.docx]

**Supporting information for:**

**“Enhancing menaquinone-7 biosynthesis by adaptive evolution of *Bacillus natto* through chemical modulator”**

Bei Zhang#, Cheng Peng#, Jianyao Lu, Xuechao Hu, Lujing Ren*

College of Biotechnology and Pharmaceutical Engineering, Nanjing Tech University, No. 30 South Puzhu Road, Nanjing 211816, People’s Republic of China

*Corresponding author. Tel./fax: +86 25 58139942.

# Bei Zhang and Cheng Peng contributed equally to this work.

E-mail: [renlujing@njtech.edu.cn](mailto:renlujing@njtech.edu.cn) (LJ. Ren);

**Figure.S1** Spore diagram of original strain (left) and ALE-25-40 (right) at the late stage of the fermentation.

**Figure.S2** Venn diagram of the number of genes expressed in the original and domesticated strains.

**Figure.S3** The number of up- and down-regulated differentially expressed genes in domesticated strains compared to the original strain.

**Figure.S4** Volcano plot of expression difference between original and domesticated strains.

**Table 1** Differentially expressed genes associated with MK-7 biosynthesis.

**Table 2** Differential expression of key genes associated with spore formation.

**Table 3** Differential expression of key genes related to antioxidant defense system.

**
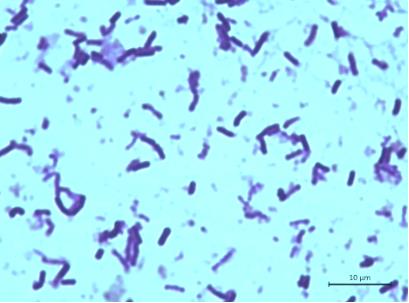

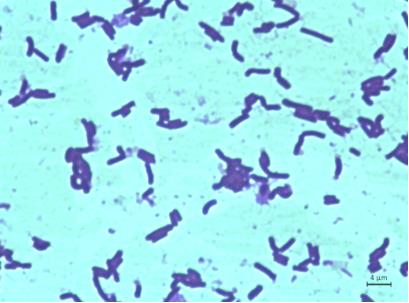
**

**Figure.S1** Spore diagram of original strain (left) and ALE-25-40 (right) at the late stage of the fermentation


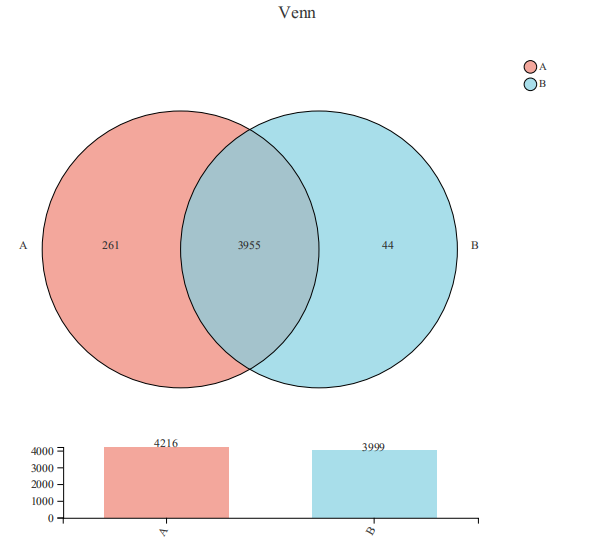


**Figure.S2** Venn diagram of the number of genes expressed in the original(A) and domesticated strains(B).


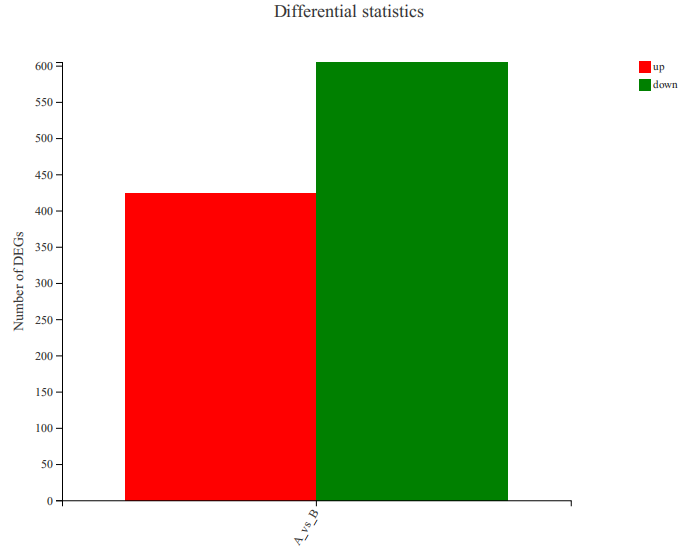


**Figure.S3** The number of up- and down-regulated differentially expressed genes in domesticated strains(B) compared to the original strain(A).


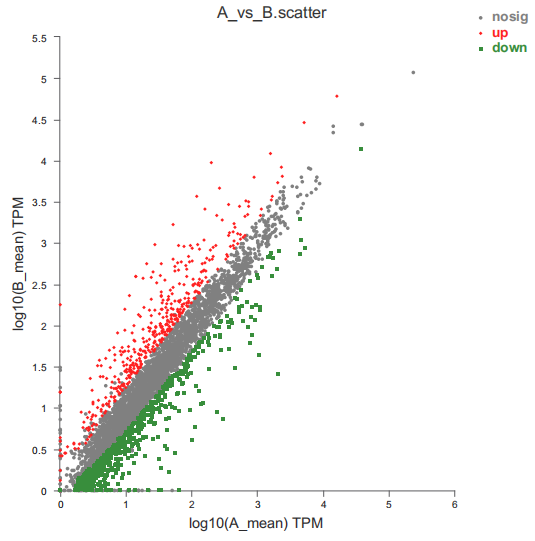


**Figure.S4** Volcano plot of expression difference between original(A) and domesticated strains(B).

**Table 1** Differentially expressed genes associated with MK-7 biosynthesis.

| Gene ID | gene_name | Description | log2FC |
| --- | --- | --- | --- |
| BSNT_07361 | glpK | glycerol kinase | 0.61 |
| BSNT_09341 | pyk | pyruvate kinase | 0.71 |
| BSNT_09342 | pfkA | 6-phosphofructokinase | 0.57 |
| BSNT_07376 | citA | citrate synthase I | -0.90 |
| BSNT_10368 | fbaA | fructose-bisphosphate aldolase | 0.28 |
| BSNT_09968 | eno | phosphopyruvate hydratase | -0.12 |
| BSNT_09394 | aroA | bifunctional 3-deoxy-7-phosphoheptulonate synthase/chorismate mutase | -0.11 |
| BSNT_08666 | aroB | 3-dehydroquinate synthase | 0.38 |
| BSNT_08711 | aroC | 3-dehydroquinate dehydratase | -0.10 |
| BSNT_09012 | aroD | shikimate 5-dehydrogenase | -0.03 |
| BSNT_06641 | aroK | shikimate kinase | 0.14 |
| BSNT_08655 | aroE | 3-phosphoshikimate 1-carboxyvinyltransferase | -0.59 |
| BSNT_08667 | aroF | chorismate synthase | -0.45 |
| BSNT_09506 | menF | menaquinone-specific isochorismate synthase | -0.14 |
| BSNT_09505 | menD | 2-succinyl-5-enolpyruvyl-6-hydroxy-3-cyclohexene-1-carboxylate synthase | -0.40 |
| BSNT_09504 | ytxM | hypothetical protein | -0.32 |
| BSNT_09501 | menC | O-succinylbenzoate-CoA synthase | -0.03 |
| BSNT_09502 | menE | O-succinylbenzoic acid--CoA ligase | -0.38 |
| BSNT_09503 | menB | naphthoate synthase | -0.34 |
| BSNT_10513 | menA | 1%2C4-dihydroxy-2-naphthoate octaprenyltransferase | -0.16 |
| BSNT_08672 | ubiE | ubiquinone/menaquinone biosynthesis methyltransferase | -0.31 |
| BSNT_08860 | dxs | 1-deoxy-D-xylulose-5-phosphate synthase | -0.57 |
| BSNT_08170 | dxr | 1-deoxy-D-xylulose 5-phosphate reductoisomerase | -0.28 |
| BSNT_06381 | ispD | 2-C-methyl-D-erythritol 4-phosphate cytidylyltransferase | -0.73 |
| BSNT_06328 | ispE | 4-diphosphocytidyl-2-C-methyl-D-erythritol kinase | -0.52 |
| BSNT_06382 | ispF | 2-C-methyl-D-erythritol 2%2C4-cyclodiphosphate synthase | -0.97 |
| BSNT_08948 | ispG | 4-hydroxy-3-methylbut-2-en-1-yl diphosphate synthase | -0.02 |
| BSNT_08957 | ispH | 4-hydroxy-3-methylbut-2-enyl diphosphate reductase | 0.67 |
| BSNT_08862 | yqiD | hypothetical protein | -0.35 |
| BSNT_08673 | hepS | heptaprenyl diphosphate synthase component I | -0.06 |

**Table 2** Differential expression of key genes associated with spore formation.

| Gene ID | gene_name | Description | log2FC |
| --- | --- | --- | --- |
| BSNT_07892 | kinA | two-component sensor histidine kinase | 0.23 |
| BSNT_09607 | kinB | two-component sensor histidine kinase | 0.16 |
| BSNT_07947 | kinC | two-component sensor histidine kinase | -0.48 |
| BSNT_07857 | kinD | two-component sensor histidine kinase | -0.55 |
| BSNT_07843 | kinE | two-component sensor histidine kinase | 0.07 |
| BSNT_08855 | spo0A | two-component response regulator | -0.27 |
| BSNT_09196 | spo0B | sporulation initiation phosphotransferase | 0.04 |
| BSNT_10369 | spo0F | two-component response regulator | -0.07 |
| BSNT_07855 | spo0E | negative sporulation regulatory phosphatase | -0.63 |
| BSNT_10404 | rapF | response regulator aspartate phosphatase | 0.64 |
| BSNT_07719 | phrA | inhibitor of the activity of phosphatase RapA | -2.03 |
| BSNT_10320 | rapB | response regulator aspartate phosphatase | 0.39 |
| BSNT_07718 | rapA | response regulator aspartate phosphatase | -1.77 |
| BSNT_08128 | codY | transcriptional repressor CodY | -2.32 |
| BSNT_08898 | sinR | transcriptional regulator | -0.60 |
| BSNT_10022 | slr | transcriptional regulator | 0.23 |
| BSNT_08897 | sinI | antagonist of SinR | -0.24 |

**Table 3** Differential expression of key genes related to antioxidant defense system.

| Gene ID | gene_name | Description | log2FC |
| --- | --- | --- | --- |
| BSNT_08944 | sodA | superoxide dismutase | 0.62 |
| BSNT_08496 | sodF | superoxide dismutase | 1.05 |
| BSNT_07288 | katA | vegetative catalase 1 | 1.78 |
| BSNT_08579 | bsaA | glutathione peroxidase | 0.26 |
| BSNT_10699 | ahpF | alkyl hydroperoxide reductase large subunit and NADH dehydrogenase | -0.04 |
| BSNT_09804 | mrgA | metalloregulation DNA-binding stress protein | -0.03 |
